# Discovery of Benzo[*f*]indole-4,9-dione Derivatives as New Types of Anti-Inflammatory Agents

**DOI:** 10.3390/ijms16036532

**Published:** 2015-03-23

**Authors:** You-Ren Chen, Chih-Hua Tseng, Yeh-Long Chen, Tsong-Long Hwang, Cherng-Chyi Tzeng

**Affiliations:** 1 Department of Medicinal and Applied Chemistry, College of Life Science, Kaohsiung Medical University, Kaohsiung 807, Taiwan; E-Mails: u98850003@cc.kmu.edu.tw (Y.-R.C.); yeloch@kmu.edu.tw (Y.-L.C.); 2 School of Pharmacy, College of Pharmacy, Kaohsiung Medical University, Kaohsiung 807, Taiwan; E-Mail: chihhua@kmu.edu.tw; 3 Graduate Institute of Natural Products, School of Traditional Chinese Medicine, College of Medicine, and Chinese Herbal Medicine Research Team, Healthy Aging Research Center, Chang Gung University, Taoyuan 333, Taiwan; 4 Immunology Consortium, Chang Gung Memorial Hospital, Kweishan, Taoyuan 333, Taiwan

**Keywords:** benzo[*f*]indole-4,9-dione derivatives, superoxide anion generation, elastase release, anti-inflammatory agents

## Abstract

Certain benzo[*f*]indole-4,9-dione derivatives were synthesized and evaluated for their inhibitory effects on superoxide anion generation and neutrophil elastase (NE) release in formyl-l-methionyl-l-leucyl-l-phenylalanine (fMLF)-activated human neutrophils. Results indicated that (*Z*)-1-benzyl-4-(hydroxyimino)-1*H*-benzo[*f*]indol-9(4*H*)-one (**10**) showed a potent dual inhibitory effect on NE release and superoxide anion generation with IC_50_ value of 2.78 and 2.74 μM respectively. The action mechanisms of **10** in human neutrophils were further investigated. Our results showed that compound **10** did not alter fMLF-induced phosphorylation of Src (Src family Y416). Notably, phosphorylation of Akt (S473) and mobilization of [Ca^2+^]_i_ caused by fMLF was inhibited by compound **10**. Further structural optimization of **10** is ongoing.

## 1. Introduction

Human neutrophils play an important role in the defense system against invasion by microorganisms and in the pathogenesis of various diseases such as rheumatoid arthritis, ischemia-reperfusion injury, chronic obstructive pulmonary disease, and asthma [1,2,3,4,5]. In response to diverse stimuli, activated neutrophils secrete a series of cytotoxins, such as superoxide anion, a precursor of other reactive oxygen species (ROS), granule proteases, and bioactive lipids [2,6,7]. Of these, neutrophil elastase (NE) is stored in the azurophil granules of neutrophils and is released following neutrophil exposure to inflammatory stimuli. High concentrations of ROS and NE have been implicated in the pathogenesis of many acute and chronic pulmonary diseases including asthma, chronic obstructive pulmonary disease, cystic fibrosis, and acute respiratory distress syndrome [2,8,9,10]. Therefore, inhibition of neutrophils activation and the following release of inflammatory mediators provide a promising strategy for the development of potential anti-inflammatory agents.

**Figure 1:**
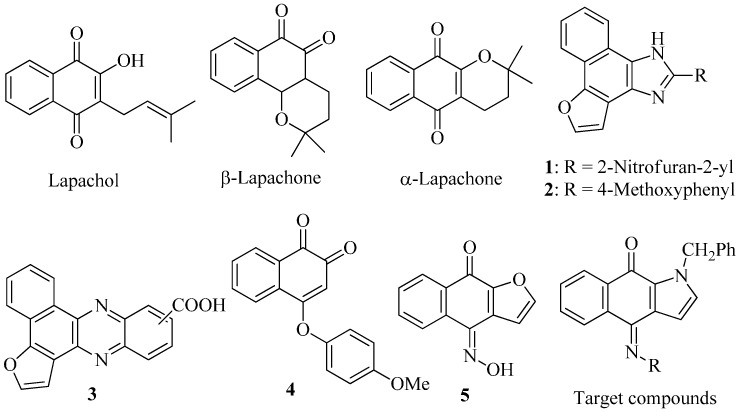
Structures of lapachol, β-lapachone, α-lapachone, and compounds **1**–**5**.

Many efforts have been devoted to the discovery of novel anti-inflammatory agents for the past few years [11,12,13,14,15,16]. The natural quinones including lapachol, α-lapachone, and β-lapachone (β-LAPA) (Figure 1) were isolated from the heartwood of the Bignoniaceae family (*Tabebuia* sp.) and evaluated for their biological activities. Among them, β-LAPA was found to be able to inhibit the expression of nitric oxide (NO) and PGE_2_ in alveolar macrophages [17]. In order to discover novel drug candidates, we have synthesized certain furo[3',2':3,4]naphtho[1,2-*d*]imidazole derivatives and evaluated for their anti-inflammatory activities. Our results indicated that (*E*)-2-[2-(5-nitrofuran-2-yl)vinyl)]-furo[3',2':3,4]naphtho[1,2-*d*]imidazole (1) [18] was capable of inhibiting iNOS expression, with an IC_50_ value of 0.52 μM while 2-(4-methoxyphenyl)furo[3',2':3,4]naphtho[1,2-*d*]imidazole (**2**) [19] exhibited a strongly inhibitory activity on LPS-induced PGE_2_ production, with an IC_50_ value of 0.047 μM. We have also demonstrated that benzo[*a*]furo[2,3-*c*]phenazinecarboxylic acid (**3**) [20] strongly inhibited superoxide anion generation while 4-(4-methoxyphenoxy)naphthalene-1,2-dione (**4**) [21] was able to inhibit NO and TNF-α released in LPS-induced Raw 264.7 cells. In continuation of our search for novel type of anti-inflammatory agents, the present study describes preparation and biological evaluation of certain benzo[*f*]indole-4,9-dione derivatives which belong to a new structural type possessing versatile iminoquinone moiety. Their cytotoxicities were also evaluated due to the structural similarity of these tricyclic compounds to the cytotoxic (*Z*)-4-(hydroxyimino)naphtho[2,3-*b*]furan-9(4H)-one (**5**) [22] which exhibited an IC_50_ value of 0.82 μM against the growth of K562 cell.

## 2. Results and Discussion

### 2.1. Chemistry

**Scheme 1:**
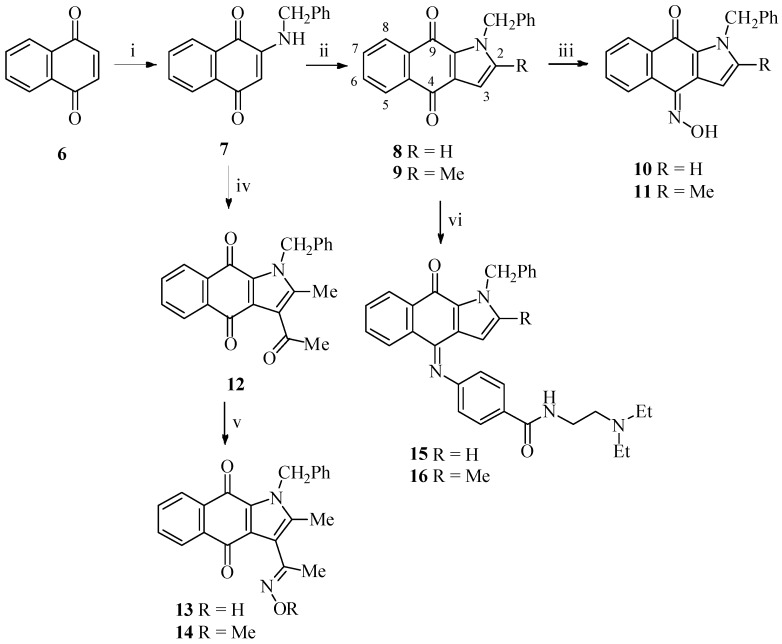
Synthesis of benzo[*f*]indole-4,9-dione derivatives **8**–**16**.

Treatment of naphthalene-1,4-dione (**6**) with benzylamine afforded 2-(benzylamino)naphthalene-1,4-dione (**7**) [23] in 78% yield as described in Scheme 1. Condensation of **7** with acetaldehyde or acetone afforded 1-benzyl-1*H*-benzo[*f*]indole-4,9-dione (**8**) and 1-benzyl-2-methyl-1*H*-benzo[*f*]indole-4,9-dione (**9**) respectively in a moderate yield. Treatment of **8** with NH_2_OH proceeds in the regiospecific and the stereospecific manners to give (*Z*)-1-benzyl-4-(hydroxyimino)-1*H*-benzo[*f*]indol-9(4*H*)-one (**10**) as a sole product. The regiospecific oximination occurred at C-4 rather than C-9 carbonyl was established based on the ^13^C-NMR in which the more downfield C-4 carbonyl shifted from 180.98 to 141.42 ppm while the more upfield C-9 carbonyl shifted from 176.23 to 174.12 ppm [22,24]. The stereospecific oximination to give *Z*-form product rather than the *E*-isomer can be realized in which the hydroxyl group proximate the pyrrole ring is less sterically hindered [25]. Accordingly, compound **11** was prepared from **9** with NH_2_OH.

The known 3-acetyl-1-benzyl-2-methyl-1*H*-benzo[*f*]indole-4,9-dione (**12**) [26] was obtained by the reaction of **7** with acetylacetone. Treatment of **12** with NH_2_OH or NH_2_OMe gave 1-benzyl-3-[1-(hydroxyimino)ethyl]-2-methyl-1*H*-benzo[*f*]indole-4,9-dione (**13**) and its methoxyimino analog **14** respectively. Reaction of **8** and **9** with procainamide proceeds in the regiospecific and the stereospecific manners to afford (*Z*)-4-(1-benzyl-9-oxo-1*H*-benzo[*f*]indol-4(9*H*)-ylideneamino)-*N*-[2-(diethylamino)ethyl]benzamide (**15**) and (*Z*)-4-(1-benzyl-2-methyl-9-oxo-1*H*-benzo[*f*]indol-4(9*H*)-ylideneamino)-*N*-[2-(diethylamino)ethyl]benzamide (**16**) respectively in a fairly good overall yield. The formation of *Z*-form product rather than the *E*-isomer can be realized in which the 4-substituted phenyl group proximate the pyrrole ring is less sterically hindered [22,25].

### 2.2. Biological Results and Discussion

Certain benzo[*f*]indole-4,9-dione derivatives were synthesized and evaluated for their inhibitory effects on superoxide anion generation and neutrophil elastase (NE) release in formyl-l-methionyl-l-leucyl-l-phenylalanine (fMLF)-activated human neutrophils and results are shown in Table 1. 1-Benzyl-1*H*-benzo[*f*]indole-4,9-dione (**8**) and its 2-methyl derivative **9** exhibited weak inhibitory effects on superoxide anion generation and were inactive on the inhibition of NE release. (*Z*)-1-Benzyl-4-(hydroxyimino)-1*H*-benzo[*f*]indol-9(4*H*)-one (**10**) showed a potent dual inhibitory effect on NE release and superoxide anion generation with IC_50_ value of 2.78 and 2.74 μM respectively. In contrast, its 2-methyl derivative **11** exhibited only marginal activity on superoxide anion generation and was inactive on the inhibition of NE release. These results indicated that the oxime moiety enhanced anti-inflammatory activities while methyl substituent at C-2 position was unfavorable especially in the inhibition of NE release. The same structure-activity relationships were observed in which 3-acetyl-1-benzyl-2-methyl-1*H*-benzo[*f*]indole-4,9-dione (**12**) was inactive while its oxime derivative **13** exhibited a weak dual inhibitory effect on NE release and superoxide anion generation. Compound **14**, the methyl derivative of **13**, was inactive. Among these benzo[*f*]indole-4,9-dione derivatives, (*Z*)-4-(1-benzyl-9-oxo-1*H*-benzo[*f*]indol-4(9*H*)-ylideneamino)-*N*-[2-(diethylamino)ethyl]benzamide (**15**) was the most potent dual inhibitor on NE release and superoxide anion generation with IC_50_ value of 0.51 and 2.05 μM respectively. Although (*Z*)-4-(1-benzyl-2-methyl-9-oxo-1*H*-benzo[*f*]indol-4(9*H*)-ylideneamino)-*N*-[2-(diethylamino)ethyl]benzamide (**16**) exhibited a strong inhibitory effect on superoxide anion generation with an IC_50_ value of 0.52 μM, it induced NE release of human neutrophils.

Reagents and conditions: i) benzylamine, EtOH, reflux; ii) MeCHO or Me_2_CO, Mn(Ac)_3_, iii) NH_2_OHHCl, K_2_CO_3_, EtOH; iv) acetylacetone, CAN; v) NH_2_OR-HCl (R = H or Me), K_2_CO_3_, EtOH; vi) procainamide, TiCl_4_, CH_2_Cl_2_.

**Table 1:** Anti-inflammatory activities of benzo[*f*]indole-4,9-dione derivatives in formyl-l-methionyl-l-leucyl-l-phenylalanine (fMLF)-activated human neutrophils (IC_50_ in μM) ^a^.

Compound	Superoxide Anion	Elastase Release
**8**	16.10 ± 1.87	>30
**9**	14.04 ± 4.40	>30
**10**	2.78 ± 0.89	2.74 ± 1.20
**11**	7.47 ± 1.39	>30
**12**	>30	>30
**13**	10.54 ± 0.52	14.65 ± 2.44
**14**	>30	>30
**15**	0.51 ± 0.12	2.05 ± 0.21
**16**	0.52 ± 0.11	– ^b^
**LY294002 ^c^**	1.36 ± 0.33	2.21±0.45

^a^ Concentration necessary for 50% inhibition (IC_50_); Results are presented as mean ± SEM (*n =* 3); ^b^ Alone induced elastase release of human neutrophils; and ^c^ LY294002 (a phosphatidylinositol-3-kinase inhibitior) was used as a positive control for superoxide anion generation and elastase release.

These benzo[*f*]indole-4,9-dione derivatives were evaluated *in vitro* against a panel of cell lines consisting of MCF7 (breast), NCI-H460 (lung), and SF-268 (CNS) as described previously [27]. Compounds which reduced the growth of any one of the cell lines to 50% or less at the concentration of 4 μg/mL were considered cytotoxic and subjected to further evaluation for their dose–response effects and IC_50_ measurement. Results from Table 2 indicated compounds **15** and **16** were cytotoxic and their IC_50_ against three cancer cells and a normal cell, Detroit 551, were shown in Table 3. The IC_50_ value of compounds **15** and **16** ranged between 3.16 and 27.41 μM and were much less cytotoxic than that of camptothecin (CPT).

**Table 2:** Cytotoxicity (% survival rate) of benzo[*f*]indole-4,9-dione derivatives at 4 μg/mL.

Cells\Compd	8	9	10	11	12	13	14	15	16	CPT ^a^
MCF7	99	105	110	104	78	106	104	82	57	33
NCI-H460	96	104	110	110	88	111	108	31	18	1
SF-268	103	106	106	102	115	109	112	120	106	25

^a^ CPT: camptothecin.

**Table 3:** Inhibition of *in vitro* cancer cell lines by benzo[*f*]indole-4,9-dione derivatives [IC_50_ (μM) ± standard deviation] ^a^.

Compd\Cells	MCF7	NCI-H460	SF-268	Detroit 551
**15**	4.98 ± 0.15	3.16 ± 0.13	27.41 ± 0.39	7.68 ± 0.58
**16**	4.95 ± 0.21	3.23 ± 0.34	25.76 ± 1.66	4.78 ± 0.57
**CPT ^b^**	0.57 ± 0.03	0.03 ± 0.003	0.19 ± 0.006	0.99 ± 0.09

^a^ Values representative mean ± standard deviation from three experiments; and ^b^ CPT: camptothecin.

**Figure 2:**
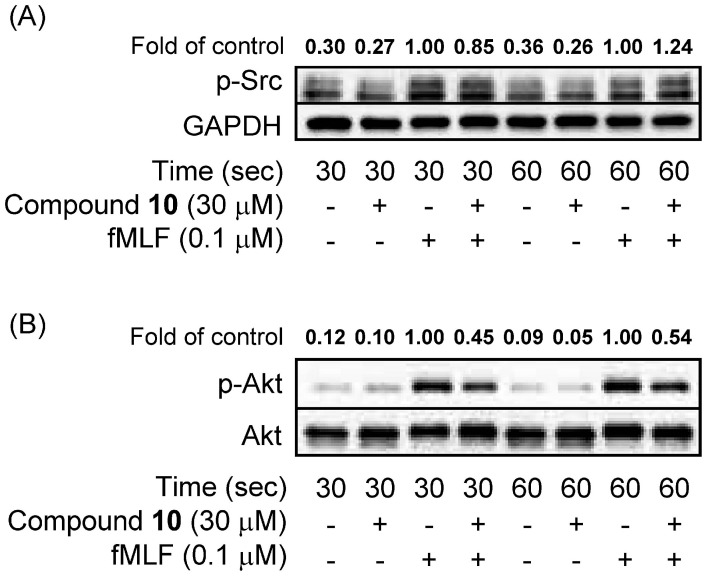
Compound **10** inhibits phosphorylation of Akt, but not Src, in fMLF-activated human neutrophils. Quantitation of the p-Scr/GADPH (**A**) and p-Akt/Akt (**B**) ratios is shown. Representative images from one of three experiments are shown.

**Figure 3:**
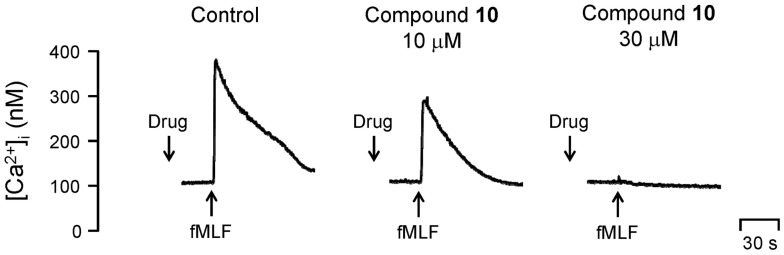
Compound **10** inhibits fMLF-induced [Ca^2+^]_i_ increase in human neutrophils. The traces shown are from three different experiments.

Compound **15** (3, 10 and 30 μM) showed cytotoxicity effects in human neutrophils, as measured by lactate dehydrogenase (LDH) release. In contrast, compound **10**, even at high concentration of 30 μM, showed no cytotoxicity effects in human neutrophils (data not shown). The action mechanisms of **10** in human neutrophils were further investigated. Human neutrophil activations, such as respiratory burst and degranulation, are regulated by Akt and calcium signal pathways [28,29]. Therefore, calcuim and Akt are considered as therapeutic target for developing anti-inflammatory agents. Compound **10** did not alter fMLF-induced phosphorylation of Src (Src family Y416) (Figure 2A). Notably, phosphorylation of Akt (S473) and mobilization of [Ca^2+^]_i_ caused by fMLF was inhibited by compound **10** (Figure 2B and Figure 3).

## 3. Experimental Section

### 3.1. General

TLC: Precoated (0.2 mm) silica gel 60 F_254_ plates from EM Laboratories, Inc. (Darmstadt, Germany); Detection by UV light (254 nm). All chromatographic separations were performed using silica gel (Merck 60 230–400 mesh, Darmstadt, Germany). M.p.: Yamato MP-21 melting-point apparatus (Yamato Scientific Co., Tokyo, Japan); Uncorrected. ^1^H and ^13^C NMR spectra: Varian-Unity-400 spectrometer at 400 and 100 MHz (Varian Inc., Palo Alto, CA, USA), chemical shifts in ppm with SiMe_4_ as an internal standard (=0 ppm), coupling constants *J* in Hz. Mass spectra (HRMS) were recorded on Finnigan/Thermo Quest MAT 95XL (ThermoQuest Finnigan, Bremen, Germany). Elemental analyses were carried out on a Heraeus CHN-O-Rapid elemental analyzer (Austin, TX, USA), and results were within ±0.4% of calculated values.

#### 3.1.1. 2-(Benzylamino)naphthalene-1,4-dione (**7**)

To a stirred solution of naphthalene-1,4-dione (**6**, 0.16 g, 1.0 mmol) in EtOH (100 mL) was added benzylamine (0.31 g, 3.0 mmol) and refluxed for 12 h (TLC monitoring). The resulting solution was concentrated in vacuo and the residue thus obtained was purified by flash chromatography on silica gel, using hexane/CH_2_Cl_2_ (1/1) as eluent and crystallized from MeOH to give 0.21 g (80%) of **7** as a red solid. M.p.: 159–160 °C (lit. 160–161 °C) [23]. ^1^H-NMR (400 MHz, CDCl_3_): 4.38 (d, 2H, *J* = 6.0 Hz), 5.79 (s, 1H), 6.23 (br s, 1H, NH), 7.31–7.40 (m, 5H), 7.62 (ddd, 1H, *J* = 7.6, 7.2, 1.6 Hz), 7.73 (ddd, 1H, *J* = 7.6, 7.6, 1.2 Hz), 8.04–8.10 (m, 2H). ^13^C-NMR (100 MHz, CDCl_3_): 46.77, 101.71, 126.19, 126.26, 127.61 (2C), 128.11, 128.99 (2C), 130.46, 132.05, 133.50, 134.76, 135.84, 147.69, 181.832, 183.07.

#### 3.1.2. 1-Benzyl-1*H*-benzo[*f*]indole-4,9-dione (**8**)

A mixture of **7** (0.19 g, 1.0 mmol), acetaldehyde (0.22 g, 5.0 mmol) and 1.34 g (5.0 mmol) of Mn(OAc)_3_ in acetic acid (20 mL) was heated at 80 °C for 16 h (by TLC monitoring). The reaction mixture was diluted with 100 mL of ethyl acetate and washed with H_2_O (3 × 50 mL), brine (50 mL), dried over anhydrous MgSO_4_, and evaporated in vacuo. The residue was purified by flash chromatography on silica gel, using a gradient of ethyl acetate/hexane (1/15 to 1/12) as eluent and crystallized from MeOH to give 0.15 g (54%) of **8** as a yellow solid. M.p.: 173–174 °C. ^1^H-NMR (400 MHz, CDCl_3_): 5.71 (s, 2H, CH_2_), 6.79 (d, 1H, *J* = 2.8 Hz, 3-H), 6.99 (d, 1H, *J* = 2.8 Hz, 2-H), 7.25–7.37 (m, 5H, Ar-H), 7.65–7.69 (m, 2H, 6- & 7-H), 8.12–8.19 (m, 2H, 5- & 8-H). ^13^C-NMR (100 MHz, CDCl_3_): 52.36 (CH_2_), 108.27 (3-C), 126.49 (2-C), 126.59 (Ar-C), 127.46 (2Ar-C), 128.13 (5-C), 128.91 (2Ar-C), 129.04, 130.37, 130.82 (8-C), 133.06 (6-C), 133.11 (7-C), 133.73, 133.99, 136.39 (Ar-C), 176.23 (9-C), 180.98 (4-C). Anal. calcd for C_19_H_13_NO_2_: C 79.43, H 4.56, N 4.88; found: C 79.49, H 4.55, N 4.88.

#### 3.1.3. 1-Benzyl-2-methyl-1*H*-benzo[*f*]indole-4,9-dione (**9**)

This was prepared from **7** as described in the synthesis of **8** from **6** with acetone (0.29 g, 5 mmol) instead of acetaldehyde, to give **9** as a yellow solid (crystallized from MeOH) in a 58% yield. M.p.: 139–140 °C. ^1^H-NMR (400 MHz, CDCl_3_): 2.27 (s, 3H, 2-Me), 5.78 (s, 2H, CH_2_), 6.60 (s, 1H, 3-H), 7.06–7.08 (m, 2H, Ar-H), 7.23–7.33 (m, 3H, Ar-H), 7.63–7.67 (m, 2H, 6- & 7-H), 8.09–8.17 (m, 2H, 5- & 8-H). ^13^C-NMR (100 MHz, CDCl_3_): 12.32 (2-Me), 48.67 (CH_2_), 107.80 (3-C), 126.19 (2Ar-C), 126.38 (Ar-C), 126.45 (5-C), 127.57 (8-C), 128.57, 128.86 (2Ar-C), 130.21, 132.81 (6-C), 133.02 (7-C), 133.46, 134.21, 136.35 (2-C), 140.23 (Ar-C), 175.48 (9-C), 181.19 (4-C). Anal. calcd for C_20_H_15_NO_2_: C 79.72, H 5.02, N 4.65; found: C 79.71, H 5.03, N 4.64.

#### 3.1.4. 1-Benzyl-4-(hydroxyimino)-1*H*-benzo[*f*]indol-9(4*H*)-one (**10**)

To a suspension of **8** (0.29 g, 1.0 mmol) in 2-ethoxyethanol (30 mL) was added hydroxylamine hydrochloride (0.20 g, 3.0 mmol). After reflux for 8 h (by TLC monitoring), the cooled mixture was evaporated in vacuo and the residue was poured into H_2_O (20 mL). The crude product was purified by flash chromatography on silica gel, using MeOH/CH_2_Cl_2_ (1/20) as eluent and crystallized from MeOH to give 0.22 g (73%) of **10** as a yellow solid. M.p.: 202–203 °C. ^1^H-NMR (400 MHz, DMSO-*d*_6_): 5.80 (s, 2H, CH_2_), 7.21–7.34 (m, 6H, 3-H and Ar-H), 7.57–7.69 (m, 3H, 2-, 6- & 7-H), 8.11–8.14 and 8.28–8.31 (m, 2H, 5- & 8-H), 12.76 (s, 1H, NOH). ^13^C-NMR (100 MHz, DMSO-*d*_6_): 51.23 (CH_2_), 111.54 (3-C), 121.71, 123.22 (2-C), 125.62, 125.76 (Ar-C), 126.93 (2Ar-C), 127.42 (5-C), 128.57 (2Ar-C), 128.91 (8-C), 131.26, 132.00 (6-C), 132.21 (7-C), 133.96, 138.28 (Ar-C), 141.42 (4-C), 174.12 (9-C). Anal. calcd for C_19_H_14_N_2_O_2_∙0.1H_2_O: C 75.04, H 4.71, N 9.21; found: C 74.98, H 4.69, N 9.24.

#### 3.1.5. 1-Benzyl-4-(hydroxyimino)-2-methyl-1*H*-benzo[*f*]indol-9(4*H*)-one (**11**)

This was prepared from **9** as described in the synthesis of **10** from **8**, to give **11** as a yellow solid (crystallized from EtOH) in a 73% yield. M.p.: 259–260 °C. ^1^H-NMR (400 MHz, DMSO-*d*_6_): 2.28 (s, 3H, 2-Me), 5.88 (s, 2H, CH_2_), 7.02–7.04 (m, 2H, Ar-H), 7.16 (s, 1H, 3-H), 7.23–7.34 (m, 3H, ar-H), 7.57–7.68 (m, 2H, 6- & 7-H), 8.10–8.12 and 8.28–8.30 (m, 2H, 5- & 8-H), 12.80 (s, 1H, NOH). ^13^C-NMR (100 MHz, DMSO-*d*_6_): 11.92 (2-Me), 47.80 (CH_2_), 111.73 (3-C), 121.40, 123.22 (5-C), 125.86 (Ar-C), 126.20 (2Ar-C), 126.26, 127.29 (8-C), 128.83 (2Ar-C), 129.02 (6-C), 131.64, 131.88 (7-C), 133.66, 137.78 (2-C), 140.57 (Ar-C), 141.56 (4-C), 173.48 (9-C). Anal. calcd for C_20_H_16_N_2_O_2_∙0.6H_2_O: C 73.43, H 5.30, N 8.56; found: C 73.42, H 5.10, N 8.16.

#### 3.1.6. 3-Acetyl-1-benzyl-2-methyl-1*H*-benzo[*f*]indole-4,9-dione (**12**)

To a solution of **7** (0.26 g, 1.0 mmol), acetylacetone (0.40 g, 4.0 mmol), and CHCl_3_ (3 mL) in MeOH (20 mL) was added four times with CAN (0.41 g × 4, 3.0 mmol) at 10 min intervals. The result mixture was stirred at room temperature for another 10 min and then diluted with ethyl acetate (100 mL), washed with H_2_O (50 mL × 3), brine (50 mL), dried over anhydrous MgSO_4_, and evaporated in vacuo. The residue was purified by flash chromatography on silica gel, using a gradient of MeOH/CH_2_Cl_2_ (1/100) as eluent and crystallized from MeOH to give 0.24 g (71%) of **12** as a orange solid. M.p.: 135–136 °C. (lit. 237–238 °C) [26]. ^1^H-NMR (400 MHz, CDCl_3_): 2.37 (s, 3H, 2-Me), 2.74 (s, 3H, 3-COMe), 5.84 (s, 2H, 1-NCH_2_), 7.06–7.09 (m, 2H, Ar-H), 7.25–7.35 (m, 3H, Ar-H), 7.66–7.73 (m, 2H, 6- & 7-H), 8.11–8.13 and 8.15–8.18 (m, 2H, 5- & 8-H). ^13^C-NMR (100 MHz, CDCl_3_): 10.92 (2-Me), 31.72 (Me), 48.78 (CH_2_), 123.12, 125.31, 126.23 (2Ar-C), 126.42 (Ar-C), 126.72 (5-C), 127.83 (8-C), 128.99 (2Ar-C), 129.82, 133.01, 133.25 (6-C), 133.42 (7-C), 133.53, 135.51, 142.03 (Ar-C), 176.23 (9-C), 180.74 (4-C), 199.23 (C=O). Anal. calcd for C_22_H_17_NO_3_: C 76.96, H 4.99, N 4.08; Found: C 77.01, H 4.99, N 4.42.

#### 3.1.7. 1-Benzyl-3-[1-(hydroxyimino)ethyl]-2-methyl-1*H*-benzo[*f*]indole-4,9-dione (**13**)

This was prepared from **12** as described in the synthesis of **10** from **8**, to give **13** as a yellow solid (crystallized from MeOH) in a 78% yield. M.p.: 204–205 °C. ^1^H-NMR (400 MHz, DMSO-*d*_6_): 2.12 (s, 3H, 2-Me), 2.21 (s, 3H, 3-C(=N)Me), 5.84 (s, 2H, 1-NCH_2_), 7.12–7.14 (m, 2H, Ar-H), 7.26–7.37 (m, 3H, Ar-H), 7.77–7.81 (m, 2H, 6- & 7-H), 8.01–8.04 (m, 2H, 5- & 8-H), 11.09 (s, 1H, NOH). ^13^C-NMR (100 MHz, DMSO-*d*_6_): 10.17 (2-Me), 16.03 (Me), 48.29 (CH_2_), 119.87, 124.85, 125.98 (5-C), 126.03 (Ar-C), 126.33 (2Ar-C), 127.45 (8-C), 128.83 (2Ar-C), 129.21, 132.94, 133.28, 133.46 (6-C), 133.53 (7-C), 136.48, 139.01, 149.88 (C=N), 174.78 (9-C), 179.87 (4-C). Anal. calcd for C_22_H_18_N_2_O_3_: C 73.73, H 5.06, N 7.82; Found: C 73.56, H 5.04, N 7.76.

#### 3.1.8. 1-Benzyl-3-[1-(methoxyimino)ethyl]-2-methyl-1*H*-benzo[*f*]indole-4,9-dione (**14**)

This was prepared from **12** as described in the synthesis of **10** from **8** and *O*-methylhydroxylamine hydrochloride instead of hydroxylamine hydrochloride, to give **14** as a yellow solid (crystallized from MeOH) in a 79% yield. M.p.: 168–169 °C. ^1^H-NMR (400 MHz, DMSO-*d*_6_): 2.13 (s, 3H, 2-Me), 2.26 (s, 3H, 3-C(=N)Me), 3.88 (s, 3H, NOMe), 5.84 (s, 2H, 1-NCH_2_), 7.13–7.15 (m, 2H, Ar-H), 7.27–7.36 (m, 3H, Ar-H), 7.79–7.82 (m, 2H, 6- & 7-H), 8.02–8.05 (m, 2H, 5- & 8-H). ^13^C-NMR (100 MHz, DMSO-*d*_6_): 10.04 (2-Me), 16.68, 48.29, 61.32, 118.66, 124.85, 126.01 (5-C), 126.08 (Ar-C), 126.38 (2Ar-C), 127.47 (8-C), 128.84 (2Ar-C), 129.36, 132.87, 133.25, 133.53 (6-C), 133.61 (7-C), 136.39, 139.09, 151.19 (C=N), 174.86 (9-C), 179.85 (4-C). Anal. calcd for C_23_H_20_N_2_O_3_: C 74.18, H 5.41, N 7.52; Found: C 74.15, H 5.39, N 7.35.

#### 3.1.9. 4-(1-Benzyl-9-oxo-1*H*-benzo[*f*]indol-4(9*H*)-ylideneamino)-*N*-[2-(diethylamino)ethyl]benzamide (**15**)

To a vigorously stirred solution of **8** (0.29 g, 1.0 mmol) in dry dichloromethane (20 mL) at room temperature was added a 1.0 M solution titanium tetrachloride in dichloromethane (1.0 mL, 1.0 mmol). To the resulting violet solution was added a solution of the procainamide (1.2 g, 5.0 mmol) in dichloromethane (10 mL) followed immediately by dry triethylamine (1.78 mL, 12.4 mmol). Two portions of 1.0 M solution of titanium tetrachloride in dichloromethane (2.0 mL, 2.0 mmol each) were added at 30 min intervals, and then the reaction mixture was poured over 100 mL of cold water and extracted with dichloromethane. The organic layer was dried with MgSO_4_ and evaporated in vacuo. The crude product was chromatographed on a column of silica gel using CH_2_Cl_2_/MeOH (10/1) to give 0.34 g (67%) of **15** as a brown solid. M.p.:152–153 °C. ^1^H-NMR (400 MHz, CDCl_3_): 1.36 (t, 6H, *J* = 7.2 Hz, NCH_2_CH_3_), 3.07 (q, 4H, *J* = 7.2 Hz, NCH_2_CH_3_), 3.17 (t, 2H, *J* = 5.2 Hz, NHCH_2_CH_2_N), 3.85 (q, 2H, *J* = 5.2 Hz, NHCH_2_CH_2_N), 5.32 (d, 1H, *J* = 2.8 Hz, 3-H), 5.68 (s, 2H, CH_2_), 6.67 (d, 1H, *J* = 2.8 Hz, 2-H), 6.94 (d, 2H, *J* = 8.8 Hz, Ar-H), 7.17–7.32 (m, 5H, Ar-H), 7.60–7.68 (m, 2H, 7- & 8-H), 8.09 (d, 2H, *J* = 8.8 Hz, Ar-H), 8.21 (dd, 1H, *J* = 7.6, 0.8 Hz, 5-H), 8.47 (br s, 1H, NH), 8.50 (dd, 1H, *J* = 7.2, 0.8 Hz, 8-H). ^13^C-NMR (100 MHz, CDCl_3_): 9.36 (2C), 35.88, 48.15 (CH_2_), 52.26 (2C), 52.94, 110.21 (3-C), 117.72 (2Ar-C), 123.76, 126.07 (5-C), 126.45 (8-C), 127.30 (Ar-C), 127.85, 128.29, 128.53 (2Ar-C), 128.76 (2Ar-C), 129.13 (2Ar-C), 129.81, 130.80 (6-C), 132.39 (7-C), 132.95, 135.80, 136.77, 151.75, 156.24, 167.43 (4-C), 176.07 (9-C). Anal. calcd for C_32_H_32_N_4_O_2_∙1.9H_2_O: C 71.33, H 6.70, N 10.40; found: C 71.69, H 6.52, N 10.01.

#### 3.1.10. 4-(1-Benzyl-2-methyl-9-oxo-1*H*-benzo[*f*]indol-4(9*H*)-ylideneamino)-*N*-[2-(diethylamino)-ethyl]benzamide (**16**)

This was prepared from **9** as described in the synthesis of **15** from **8**, to give **16** as a yellow solid in a 43% yield. M.p.:138–139 °C. ^1^H-NMR (400 MHz, CDCl_3_): 1.38 (t, 6H, *J* = 7.2 Hz, NCH_2_CH_3_), 2.02 (s, 3H, 2-Me), 3.08 (q, 4H, *J* = 7.2 Hz, NCH_2_CH_3_), 3.11 (t, 2H, *J* = 4.8 Hz, , NHCH_2_CH_2_N), 3.86 (q, 2H, *J* = 4.8 Hz, NHCH_2_CH_2_N), 5.18 (s, 1H, 3-H), 5.77 (s, 2H, CH_2_), 6.94–7.01 (m, 4H, Ar-H), 7.20–7.32 (m, 4H, Ar-H), 7.58–7.66 (m, 2H, 6- & 7-H), 8.12 (d, 2H, *J* = 8.4 Hz, Ar-H), 8.19 (dd, 1H, *J* = 7.6, 1.2 Hz, 5-H ), 8.48–8.50 (m, 2H, 8-H & NH). ^13^C-NMR (100 MHz, CDCl_3_): 9.33 (2C), 12.14 (2-Me), 35.90, 48.40 (CH_2_), 48.48 (2C), 53.23, 109.80 (3-C), 117.72 (2Ar-C), 123.38, 126.07, 126.10 (2Ar-C), 126.33 (5-C), 127.35 (8-C), 127.91, 128.08, 128.76 (2Ar-C), 129.17 (2Ar-C), 130.76 (6-C), 132.12 (7-C), 133.20, 135.51, 136.77, 138.97, 151.65, 156.35, 167.47 (4-C), 175.31 (9-C). Anal. calcd for C_33_H_34_N_4_O_2_∙2.3H_2_O: C 70.77, H 6.95, N 10.00; found: C 70.56, H 6.75, N 9.74.

### 3.2. Biological Evaluation

#### 3.2.1. Preparation of Human Neutrophils 

Blood was taken from healthy human donors (20–30 years old) by venipuncture, using a protocol approved by the institutional review board at Chang Gung Memorial Hospital. Neutrophils were isolated with a standard method of dextran sedimentation prior to centrifugation in a Ficoll Hypaque gradient and hypotonic lysis of erythrocytes [30].

#### 3.2.2. Superoxide Generation and Elastase Release

Superoxide generation and elastase release were carried out according to the procedures described previously [31]. Neutrophils (6 × 10^5^/mL) were equilibrated at 37 °C for 2 min and incubated with compounds for 5 min. Neutrophils were then activated by fMLF (100 nM) in the pretreatment of cytochalasin B (1 μg/mL for superoxide generation and 0.5 μg/mL for elastase release) for 10 min. Superoxide anion production was assayed by monitoring the superoxide dismutase-inhibitable reduction of ferricytochrome *c.* Elastase release was performed using MeO-Suc-Ala-Ala-Pro-Valp-nitroanilide as the elastase substrate.

#### 3.2.3. Western Analysis

Neutrophils were pretreated with compounds for 5 min before being stimulated with fMLF at 37 °C. The reaction was stopped by adding 5 × Laemmli’s sample buffer [32,33]. Proteins derived from whole-cell lysates were separated by SDS-polyacrylamide gel electrophoresis (PAGE) using polyacrylamide gels and blotted onto nitrocellulose membranes. Immunoblotting was performed using the indicated antibodies and horseradish peroxidase (HRP)-conjugated secondary anti-rabbit antibodies (Cell Signaling Technology, Beverly, MA, USA). The immunoreactive bands were visualized by an enhanced chemiluminescence system (Amersham Biosciences, Bucks, UK) and detected by Ultraviolet Product (UVP) imaging system (Upland, CA, USA).

#### 3.2.4. Measurement of Intracellular Calcium Concentration ([Ca^2+^]_i_)

Neutrophils were loaded with fluo-3 AM (2 μM) at 37 °C for 45 min. Cells were preincubated with compounds for 5 min, and then activated by fMLF (100 nM). The change in fluorescence was monitored using a Hitachi F-4500 spectrofluorometer (Tokyo, Japan). The excitation wavelength was 488 nm, and the emission wavelength was 520 nm.

## 4. Conclusions

We have identified (*Z*)-1-benzyl-4-(hydroxyimino)-1*H*-benzo[*f*]indol-9(4*H*)-one (**10**) as a novel structural type of potential anti-inflammatory agent. Compound **10** exhibited dual inhibitory activities on NE release and superoxide anion generation in formyl-l-methionyl-l-leucyl-l-phenylalanine (fMLF)-activated human neutrophils with IC_50_ value of 2.78 and 2.74 μM respectively. Mechanism studies indicated that compound **10** did not alter fMLF-induced phosphorylation of Src (Src family Y416). Notably, phosphorylation of Akt (S473) and mobilization of [Ca^2+^]_i_ caused by fMLF was inhibited by compound **10**. Further structural optimization of **10** is ongoing.

## References

[1] Malech H.L., Gallin J.I. (1987). Current concepts: Immunology. Neutrophils in human diseases. N. Eng. J. Med..

[2] Witko-Sarsat V., Rieu P., Descamps-Latscha B., Lesavre P., Halbwachs-Mecarelli L. (2000). Neutrophils: Molecules, functions and pathophysiological aspects. Lab. Investig..

[3] Okajima K., Harada N., Uchiba M. (2002). Ranitidine reduces ischemia/reperfusion-induced liver injury in rats by inhibiting neutrophil activation. J. Pharmacol. Exp. Ther..

[4] Ennis M. (2003). Neutrophils in asthma pathophysiology. Curr. Allergy Asthma Rep..

[5] Vinten-Johansen J. (2004). Involvement of neutrophils in the pathogenesis of lethal myocardial reperfusion injury. Cardiovasc. Res..

[6] Borregaard N. (1998). The human neutrophil. Function and dysfunction. Eur. J. Haematol..

[7] Roos D., van Bruggen R., Meischl C. (2003). Oxidative killing of microbes by neutrophils. Microbes Infect..

[8] Burgos R.A., Hidalgo M.A., Figueroa C.D., Conejeros I., Hancke J.L. (2009). New potential targets to modulate neutrophil function in inflammation. Mini Rev. Med. Chem..

[9] Hsieh P.W., Hwang T.L., Wu C.C., Chang F.R., Wang T.W., Wu Y.C. (2005). The evaluation of 2,8-disubstituted benzoxazinone derivatives as anti-inflammatory and anti-platelet aggregation agents. Bioorg. Med. Chem. Lett..

[10] Cho H.Y., Kleeberger S.R. (2007). Genetic mechanisms of susceptibility to oxidative lung injury in mice. Free Radic. Biol. Med..

[11] Meng X.L., Yang J.Y., Chen G.L., Zhang L.J., Wang L.H., Li J., Wu C.F. (2008). RV09, a novel resveratrol analogue, inhibits NO and TNF-alpha production by LPS-activated microglia. Int. Immunopharmacol..

[12] Shin H.M., Lee Y.R., Chang Y.S., Lee J.Y., Kim B.H., Min K.R., Kim Y. (2006). Suppression of interleukin-6 production in macrophages by furonaphthoquinone NFD-37. Int. Immunopharmacol..

[13] Meng X.L., Yang J.Y., Chen G.L., Wang L.H., Zhang L.J., Wang S., Li J., Wu C.F. (2008). Effects of resveratrol and its derivatives on lipopolysaccharide-induced microglial activation and their structure-activity relationships. Chem. Biol. Interact..

[14] Shin E.M., Zhou H.Y., Guo L.Y., Kim J.A., Lee S.H., Merfort I., Kang S.S., Kim H.S., Kim S., Kim Y.S. (2008). Anti-inflammatory effects of glycyrol isolated from *Glycyrrhiza uralensis* in LPS-stimulated RAW264.7 macrophages. Int. Immunopharmacol..

[15] Cote B., Boulet L., Brideau C., Claveau D., Ethier D., Frenette R., Gagnon M., Giroux A., Guay J., Guiral S. (2007). Substituted phenanthrene imidazoles as potent, selective, and orally active mPGES-1 inhibitors. Bioorg. Med. Chem. Lett..

[16] Liu F.Z., Fang H., Zhu H.W., Wang Q., Yang Y., Xu W.F. (2008). Design, synthesis, and preliminary evaluation of 4-[6-(3-nitroguanidino)hexanamido]pyrrolidine derivatives as potential iNOS inhibitors. Bioorg. Med. Chem..

[17] Liu S.H., Tzeng H.P., Kuo M.L., Lin-Shiau S.Y. (1999). Inhibition of inducible nitric oxide synthase by *β*-lapachone in rat alveolar macrophages and aorta. Br. J. Pharmacol..

[18] Tseng C.H., Lin C.S., Shih P.K., Tsao L.T., Wang J.P., Cheng C.M., Tzeng C.C., Chen Y.L. (2009). Furo[3',2':3,4]naphtho[1,2-*d*]imidazole derivatives as potential inhibitors of inflammatory factors in sepsis. Bioorg. Med. Chem..

[19] Tseng C.H., Tzeng C.C., Shih P.K., Yang C.N., Chuang Y.C., Peng S.I., Lin C.S., Wang J.P., Cheng C.M., Chen Y.L. (2012). Identification of furo[3',2':3,4]naphtho[1,2-*d*]imidazole derivatives as orally active and selective inhibitors of microsomal prostaglandin E(2) synthase-1 (mPGES-1). Mol. Divers..

[20] Tsai Y.R., Huang L.J., Lee M.R., Chen Y.L., Kuo S.C., Tzeng C.C., Hsu M.F., Wang J.P. (2012). The signaling mechanisms mediating the inhibitory effect of TCH-1116 on formyl peptide-stimulated superoxide anion generation in neutrophils. Eur. J. Pharm..

[21] Tseng C.H., Cheng C.M., Tzeng C.C., Peng S.I., Yang C.L., Chen Y.L. (2013). Synthesis and anti-inflammatory evaluations of β-lapachone derivatives. Bioorg. Med. Chem..

[22] Tseng C.H., Chen Y.L., Yang S.H., Peng S.I., Cheng C.M., Han C.H., Lin S.R., Tzeng C.C. (2010). Synthesis and antiproliferative evaluation of certain iminonaphtho[2,3-*b*]furan derivatives. Bioorg. Med. Chem..

[23] Aristoff P.A., Johnson P.D. (1992). Synthesis of CBI-PDE-I-dimer, the benzannelated analog of CC-1065. J. Org. Chem..

[24] Urbanek R.A., Suchard S.J., Steelman G.B., Knappenberger K.S., Sygowski L.A., Veale C.A., Chapdelaine M.J. (2001). Potent reversible inhibitors of the protein tyrosine phosphatase CD45. J. Med. Chem..

[25] Ito C., Katsuno S., Kondo Y., Tan H.T., Furukawa H. (2000). Chemical constituents of *Avicennia alba*. Isolation and structural elucidation of new naphthoquinones and their analogues. Chem. Pharm. Bull..

[26] Tseng C.C., Wu Y.L., Chuang C.P. (2002). Cerium salts in the oxidative free radical reactions between 2-amino-1,4-naphthoquinones and β-dicarbonyl compounds. Tetrahedron.

[27] Chen Y.C., Cheng M.J., Lee S.J., Dixit A.K., Ishikawa T., Tsai I.L., Chen I.S. (2004). Coumarinolignans from the Root of Formosan Antidesma pentandrum var.barbatum. Helv. Chim. Acta.

[28] Hwang T.L., Su Y.C., Chang H.L., Leu Y.L., Chung P.J., Kuo L.M., Chang Y.J. (2009). Suppression of superoxide anion and elastase release by C_18_ unsaturated fatty acids in human neutrophils. J. Lipid Res..

[29] Hwang T.L., Wang C.C., Kuo Y.H., Huang H.C., Wu Y.C., Kuo L.M., Wu Y.H. (2010). The hederagenin saponin SMG-1 is a natural FMLP receptor inhibitor that suppresses human neutrophil activation. Biochem. Pharmacol..

[30] Boyum A., Lovhaug D., Tresland L., Nordlie E.M. (1991). Separation of leucocytes: Improved cell purity by fine adjustments of gradient medium density and osmolality. Scand. J. Immunol..

[31] Hwang T.L., Li G.L., Lan Y.H., Chia Y.C., Hsieh P.W., Wu Y.H., Wu Y.C. (2009). Potent inhibition of superoxide anion production in activated human neutrophils by isopedicin, a bioactive component of the Chinese medicinal herb *Fissistigma oldhamii*. Free Radic. Biol. Med..

[32] Gilbert C., Rollet-Labelle E., Caon A.C., Naccache P.H. (2002). Immunoblotting and sequential lysis protocols for the analysis of tyrosine phosphorylation-dependent signaling. J. Immunol. Methods.

[33] Chan H.H., Hwang T.L., Thang T.D., Leu Y.L., Kuo P.C., Nguyet B., Dai D.N., Wu T.S. (2013). Isolation and synthesis of melodamide A, a new anti-inflammatory phenolic amide from the leaves of *Melodorum fruticosum*. Planta Med..

